# EV-Associated MMP9 in High-Grade Serous Ovarian Cancer Is Preferentially Localized to Annexin V-Binding EVs

**DOI:** 10.1155/2017/9653194

**Published:** 2017-05-16

**Authors:** Agnes T. Reiner, Sisareuth Tan, Christiane Agreiter, Katharina Auer, Anna Bachmayr-Heyda, Stefanie Aust, Nina Pecha, Mattias Mandorfer, Dietmar Pils, Alain R. Brisson, Robert Zeillinger, Sai Kiang Lim

**Affiliations:** ^1^BioSensor Technologies, AIT-Austrian Institute of Technology GmbH, Muthgasse 11, 1190 Vienna, Austria; ^2^Institute of Medical Biology, A∗STAR, 8A Biomedical Grove, No. 05-05 Immunos, Singapore 138648; ^3^Molecular Oncology Group, Department of Obstetrics and Gynecology, Medical University of Vienna, Waehringer Guertel 18-20, 1090 Vienna, Austria; ^4^Extracellular Vesicles and Membrane Repair, UMR-5248-CBMN, CNRS-University of Bordeaux-IPB, Allée Geoffroy Saint-Hilaire, 33600 Pessac, France; ^5^Division of Gastroenterology and Hepatology, Department of Internal Medicine III, Medical University of Vienna, Waehringer Guertel 18-20, 1090 Vienna, Austria; ^6^Section for Clinical Biometrics, Center for Medical Statistics, Informatics, and Intelligent Systems (CeMSIIS), Medical University of Vienna, Waehringer Guertel 18-20, 1090 Vienna, Austria; ^7^Department of Surgery, Medical University of Vienna, Waehringer Guertel 18-20, 1090 Vienna, Austria

## Abstract

High-grade serous ovarian cancer (HGSOC) is the most aggressive type of ovarian cancer and is responsible for most deaths caused by gynecological cancers. Numerous candidate biomarkers were identified for this disease in the last decades, but most were not sensitive or specific enough for clinical applications. Hence, new biomarkers for HGSOC are urgently required. This study aimed to identify new markers by isolating different extracellular vesicle (EV) types from the ascites of ovarian cancer patients according to their affinities for lipid-binding proteins and analyzing their protein cargo. This approach circumvents the low signal-to-noise ratio when using biological fluids for biomarker discovery and the issue of contamination by large non-EV complexes. We isolated and analyzed three distinct EV populations from the ascites of patients with ovarian cancer or cirrhosis and observed that Annexin V-binding EVs have higher levels of matrix metalloproteinase 9 in malignant compared to portal-hypertensive ascites. As this protein was not detected in other EV populations, this study validates our approach of using different EV types for optimal biomarker discovery. Furthermore, MMP9 in Annexin V-binding EVs could be a HGSOC biomarker with enhanced specificity, because its identification requires detection of two distinct components, that is, lipid and protein.

## 1. Introduction

Ovarian cancer is the deadliest among gynecologic malignancies with a 5-year survival rate after diagnosis of 27% [1]. This poor prognosis is mostly due to the asymptomatic disease progression, resulting in diagnosis at advanced stages [2] and to the lack of reliable biomarkers. Over the last few years, extracellular vesicles (EVs) have gained a lot of interest as potential sources of biomarkers in many diseases including ovarian cancer, because the secreted vesicles and their cargo resemble their cell of origin and, additionally, EVs can be easily accessed and isolated from any of our bodily fluids [3]. The collection of bodily fluids is generally less invasive than conventional tissue biopsy. In several studies of ovarian cancer EVs, cancer-derived vesicles have been identified in blood plasma, serum, and ascites fluid of patients and they presented a unique set of cargo proteins and RNAs with great potential as new diagnostic, prognostic, and predictive biomarkers [4, 5].

However, the current methods to isolate EVs from body fluids for protein biomarkers are particularly challenging for several reasons. First, the proteome of most bodily fluids is complex and protein concentrations have a dynamic range that exceeds 10^10^. For example, the proteome of plasma or serum is dominated by several high-abundance proteins. It is estimated that of the approximate 10,000 proteins in the serum, only 21 proteins (namely, albumin; IgG; transferrin; haptoglobin; transthyretin; *α*2-macroglobulin; *α*1-antitrypsin; *α*1-acid glycoprotein; hemopexin; apolipoprotein A1 and B; AGP; lipoprotein A; factor H; ceruloplasmin; complements C4, C9, C19, and C8; and prealbumin) constitute >99% of the serum protein mass [6]. As such, any technique that aims to isolate candidate biomarkers will have to isolate the remaining 1% from the bulk of high-abundance proteins. Hence, common EV isolation methods based on biophysical parameters such as high-speed centrifugation or filtration would inevitably be contaminated with the high-abundance proteins [7, 8]. Second, in addition to proteins, many bodily fluids contain large lipoprotein complexes that are similar in size and density to EVs and thus are often copurified and mistaken as EVs by common analysis methods [9, 10]. And third, individual classes of EVs, for instance, exosomes or microvesicles, are very difficult to discriminate, because they overlap not only in their physical characteristics like size or density but also in their cargo molecules [11]. Consequently, this lack of distinguishing criteria makes the isolation of pure vesicle populations almost impossible with currently used methods. Additionally, recent publications suggest that even within the earlier defined classes of EVs, there are more subclasses of vesicles with distinct cargo and functionality [12] adding another layer of complexity.

To overcome some of these obstacles, a new strategy for vesicle isolation using proteins with high specific binding affinity for membrane lipids has been proposed. In this approach, vesicles are isolated based on their membrane lipid composition, which resolves the problem of protein contamination and introduces a new way of classifying vesicle populations. The proteins used for isolation are Annexin V (AV), Cholera Toxin B-chain (CTB), and Shiga Toxin B-chain (STB), which have high specific binding affinity for phosphatidylserine, ganglioside GM1, and globotriaosylceramide, respectively. By combining these lipid-binding ligands with magnetic particles, distinct populations of EVs that can be bound by each of the lipid ligands were purified and characterized from conditioned medium of mesenchymal stem cells [13]. This lipid-based isolation approach was also used to successfully identify protein biomarkers in EVs isolated from human plasma for the diagnosis of preeclampsia [14].

The aim of this study was to examine the potential of ascitic fluid-derived EVs as markers for ovarian cancer using this new vesicle isolation method based on specific binding of lipids present in the vesicle membrane. First, the isolation method was tested for successful isolation of EVs from ascites and then the protein cargo of different EV populations isolated from the ascitic fluid of patients with ovarian cancer and cirrhosis was analyzed in regard to the presence of cancer-associated molecules.

## 2. Material and Methods

### 2.1. Patient Samples

The ascites of patients with ovarian cancer or cirrhosis was collected at the Medical University of Vienna (Austria). Patients with ovarian cancer recruited in the time from September 2013 to January 2015 were included in the study, if ascites was present and no treatment was performed before sample collection. Patients with cirrhosis were recruited between July and August 2015 and were excluded, if cirrhosis was due to hepatitis C. All patients signed an informed consent before sample collection. This study was approved by the ethical review board of the Medical University of Vienna (number 793/2011). The ascites samples of ovarian cancer patients were collected at the first presentation of the disease, before therapy or during debulking surgery.

After collection, the ascitic fluid was centrifuged at 1000*g* for 10 min and the supernatant was centrifuged a second time at 3000*g* for 10 min to remove remaining cells and cell debris. Subsequently, the supernatant was filtered through a 0.22 *μ*m filter (Merck Millipore, USA), aliquoted, and stored at −80°C for further analysis.

### 2.2. EV Isolation with Lipid-Binding Ligands

The EV isolation procedure was performed as described earlier [13]. Briefly, 1.5 ng of biotinylated lipid-binding ligand were used per *μ*l of ascites. The ligands AV, CTB, and STB were mixed with ascites in binding buffer (10 mM HEPES, 2.5 mM CaCl_2_, 140 mM NaCl, PBS pH 7.4) and incubated for 30 minutes at room temperature (RT) with shaking. Then, 0.1 *μ*l per *μ*l ascites starting volume of prewashed Dynabeads M280 Streptavidin (Thermo Fisher Scientific, USA) were added to each reaction mix and incubated again for 30 minutes at RT with shaking. The beads were separated from the liquid with a magnet, the supernatant was discarded, and the beads were washed twice with PBS. Finally, the bead bound fraction was resuspended in PBS or lysis buffer depending on the subsequent analysis method. The starting volume of ascitic fluid for EV isolation was adjusted for each analysis method and is stated in the respective sections. As a negative control for unspecific binding to the magnetic beads, the same isolation procedure was performed without an addition of a lipid-binding ligand.

### 2.3. Western Blot

Standard western blot analysis was performed by denaturing the EV samples isolated from 250 *μ*l of ascites and loading them onto 4–12% NuPAGE Bis-Tris Gels in reducing conditions (Thermo Fisher Scientific, USA). After gel electrophoresis, the proteins were transferred to a nitrocellulose membrane. The membrane was then probed with a primary antibody followed by a HRP-conjugated secondary antibody. The primary antibody was detected using a chemiluminescent HRP-substrate (Super Signal West Solution, Thermo Fisher Scientific, USA). The chemiluminescent signals were analyzed with Image Lab software (Bio-Rad Laboratories, USA). The band intensities of each individually analyzed protein were quantified by subtracting the background signal and normalizing to the corresponding *β*-actin band intensity. Results were given as the protein to *β*-actin ratio. The primary antibodies were mouse antibodies against the human proteins *β*-actin (C4), *β*-catenin (E-5), CD71 (H68.4), CD9 (C-4), Alix (1A12), CD59 (H-7), EpCAM (0.N.276) (Santa Cruz Biotechnology Inc., USA), CD63 (H5C6) (BD Biosciences, USA), and FN1-EDA (IST-9) (abcam PLC, UK). All primary antibodies were used in a 1 : 50 dilution; only anti-FN1-EDA was diluted 1 : 500. The secondary antibody was a goat anti-mouse IgG-HRP conjugate (sc-2031, Santa Cruz Biotechnology Inc., USA) and diluted 1 : 1250.

### 2.4. Zymography and MMP9 ELISA

The presence of gelatinases in the EV isolates was analyzed using Novex Zymogram Gels (Thermo Fisher Scientific, USA) according to the manufacturer's instructions. The EV isolations from 500 *μ*l ascites and 1 *μ*l of ascites without isolation were diluted in the loading buffer and resolved on a 10% zymogram (gelatin) gel in nonreducing conditions. After protein renaturation and developing the gel over night at 37°C, the gel was stained with Coomassie Blue staining solution (40 vol% methanol, 7 vol% acetic acid, and 0.25 mg/ml Brilliant Blue R (all Sigma Aldrich, USA), in water) for 30 min at 80°C and then for 4 hours at RT with agitation. Then, the gel was washed in destaining solution (40 vol% methanol, 7 vol% acetic acid, in water) for 10 min at 80°C and RT alternately.

The obtained images were analyzed with Image Lab software (Bio-Rad Laboratories, USA). The band intensities were quantified by extracting the background-corrected volume density and normalized to the number of pixels of the analyzed band. For quantification of MMP9-levels, only the 92 kDa band was analyzed.

MMP9-levels of ascites or EV isolates were quantified by ELISA. The samples were lysed with the Mammalian Cell Extraction Kit (BioVision, USA) and subsequently analyzed with the Quantikine ELISA human MMP-9 kit (R&D, USA) that were both performed according to the manufacturer's instructions, except for the sample incubation conditions in the ELISA that were changed to overnight at 4°C.

### 2.5. Cryoelectron Microscopy and AV Gold Labeling

For cryoelectron microscopy (EM) experiments, ascites samples were labeled with 4 nm gold nanoparticles (NP) conjugated to AV as follows. An 8 *μ*l aliquot of ascites was mixed with 1 *μ*l 20 mM CaCl_2_ and 1 *μ*l 1–4 × 10^16^ NP/L AV-gold-NP and incubated for 15 min. Then, 4 *μ*l were deposited on an EM grid coated with a perforated carbon film and quickly frozen in liquid ethane. EM grids were mounted on a Gatan 626 cryoholder and transferred into a Tecnai F20 (FEI) microscope operated at 200 kV. Images were recorded with an USC1000-SSCCD camera (Gatan). For the synthesis of AV-gold-NPs, refer to Arraud et al. [15].

### 2.6. Statistical Analysis

The statistical analysis was performed in R. The 2-sided, unpaired Mann–Whitney-Wilcoxon test was used for the comparison of MMP9 levels measured by zymography or ELISA between different patient groups. The correlation between zymography and ELISA results was calculated with the Spearman method, and only the range from 0 to 100 pg of MMP9 was used. The correlation coefficient (rho), as well as *p* values, was given as results, and *p* values ≤0.05 were considered statistically significant.

## 3. Results and Discussion

### 3.1. Patient Cohort

The patient cohort comprised of 17 patients with ovarian cancer and 10 with cirrhosis, respectively. The former had a median age of 61 years (39–83 yrs) at diagnosis and recruitment to the study. The later had a median age of 66 years (53–77 yrs). The clinicopathological characteristics of the ovarian cancers—histological type, FIGO stage, and grade—were assessed by a pathologist after a routine surgical removal of the cancer tissue. All clinical details of the ovarian cancer patients are given in Table 1.

### 3.2. Isolation Method Testing

**Figure 1:**
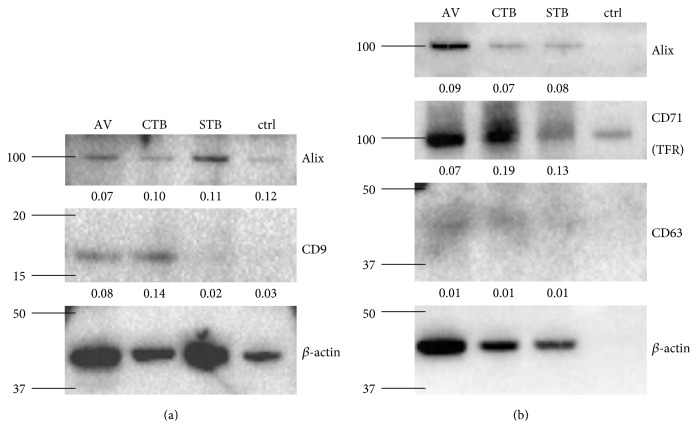
EV isolation from ascites. EVs were isolated from ascites with the ligands AV (lane 1), CTB (lane 2), and STB (lane 3) and analyzed by western blotting. As a negative control, no ligand was added (lane 4). 250 *μ*l of pooled ascites from 6 (a) or 16 (b) ovarian cancer patients were used as starting material. The tetraspanin proteins CD9 and CD63, the transferrin receptor (CD71), and the cytosolic protein Alix were examined, and *β*-actin was used as a loading control. The normalized band intensities of the analyzed proteins are given below each band. The molecular weight markers are indicated on the left side of the images in kDa.

In this study, EVs were isolated by incubating the ascites fluid with biotinylated lipid-binding protein ligands AV, CTB, and STB. Vesicles bound by these biotinylated ligands were extracted with streptavidin-coated magnetic beads and thus could be separated from proteins or other vesicles present in the sample. As this method was only used for EV isolation from conditioned cell culture medium and plasma before [13, 14], we determined if these ligands could also isolate EVs from ascitic fluid by examining the isolates for the presence of tetraspanins CD9 and CD63, the transferrin receptor or CD71, and a cytosolic protein, Alix. All these proteins are associated with EVs and generally used as EV markers [3]. *β*-actin was used as a loading control and for band intensity normalization. Due to limited sample volumes, this analysis was performed using two different pools of ascites, one pooled from 6 cancer patients (Figure 1()) and the other pooled from 16 cancer patients (Figure 1()). As shown in Figure 1(), CD9 and CD63 were detected only in the AV- and CTB-binding EVs from ascites. CD71 and Alix were detected in all three vesicle populations. As a negative control for unspecific binding of ascites proteins to the magnetic beads, the same isolation procedure was performed without the addition of a ligand (Figure 1(), lane 4). Even though some unspecific binding to the magnetic beads occurred, especially for *β*-actin and CD71, the level of the proteins in the negative control was very low compared to that in the EV isolations, suggesting specific enrichment of the tested proteins in the isolations. As CD9 and CD63 are tetraspanins with 4 membrane domains, their presence in the isolates were definitive evidence that all three isolates were lipid membrane vesicles. The different distribution profile of the four proteins relative to *β*-actin among the three EV isolates further suggested that each of the three EV isolates was unique. For example, the absence of CD9 in STB-binding EVs showed clearly that these EVs are different from both CTB-binding and AV-binding EVs [13]. Taking these results together, AV, CTB, and STB are capable of isolating unique EV populations from ascites.

### 3.3. Cancer-Marker Search

Next, AV-, CTB-, and STB-binding vesicles isolated from pooled ovarian cancer ascites or from pooled portal-hypertensive ascites were probed for the presence of cancer markers. The rationale for using ascitic fluid instead of plasma for the discovery of EV-associated ovarian cancer biomarkers is that ascites, being in direct contact with the cancer, is more likely to carry cancer biomarkers [1, 16–19]. Once candidate EV-associated ovarian cancer biomarkers are identified, we will next determine if these ascites EV-associated candidate biomarkers can be found in plasma as well. This strategy will enhance the chance of identifying ovarian cancer biomarkers where the signal-to-noise ratio will be higher than that in the plasma, where the vast majority of EVs are derived from blood cells. The following cancer-associated proteins were selected for analysis in the EV isolations based on literature search: cellular fibronectin (FN1-EDA), protectin (CD59), epithelial cell adhesion molecule (EpCAM), *β*-catenin, and gelatinases matrix metalloproteinase 2 and 9 (MMP2/9).

**Figure 2:**
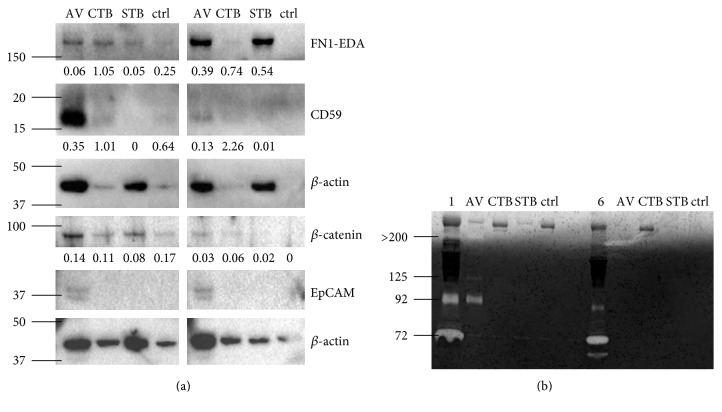
Cancer proteins in EV isolations. (a) EVs isolated from 250 *μ*l of pooled ascites from 6 ovarian cancer patients (lanes 1–4) or from 5 patients with cirrhosis (lanes 5–8) were analyzed by western blot. As before the ligands AV, CTB, and STB were used for isolation, no ligand was added as a negative control. The protein cellular fibronectin (FN1-EDA), CD59, *β*-catenin, and epithelial cell adhesion molecule (EpCAM) were examined, and *β*-actin was used as loading control. The normalized band intensities of the analyzed proteins are given below each band. (b) EVs isolated from 500 *μ*l of the same samples as described for (a) were analyzed by zymography. The left part of the image (lanes 1–5) shows the ovarian cancer samples and the right part the cirrhosis samples (lanes 6–10). In lanes 1 and 6, 1 *μ*l of the pooled ascites samples were analyzed. The molecular weight markers are indicated on the left side of the images in kDa.

FN1-EDA, also called cellular fibronectin, is a splice variant of fibronectin and an important component of the extracellular matrix. It is highly expressed during embryogenic development but barely found in adult tissues in contrast to the soluble fibronectin isotype, which is constantly produced by hepatocytes and generally present in plasma [20]. FN1-EDA was found in a variety of cancer tissues [21] and plays an important role in angiogenesis and cell migration [20] which are two processes crucial for carcinogenesis and metastasis. This protein was detected in all three EV types isolated from the ascites of patients with ovarian cancer and was highly elevated in AV- and STB-EVs of the cirrhosis patients (Figure 2). The elevated level of FN1-EDA in EVs of patients with cirrhosis is consistent with that of the previous report of its important role in experimental hepatic fibrosis [22]. However, this observation eliminated the utility of FN1-EDA as an ovarian cancer marker.

CD59 is a GPI-anchored membrane protein that is expressed on the surface of cells to protect them from complement-mediated cell lysis by inhibiting the assembly of the membrane attack complex. Many types of cancer, including ovarian cancer, express CD59 to escape the immune system [23]. Furthermore, CD59 has been found on EVs which enabled them to protect themselves and other cells from complement attack [24–26]. Interestingly, it was previously reported that CD59 is not only present in the ascites of ovarian cancer patients but is also associated with the phospholipid fraction [27]. Consistent with this, we delete detected CD59 in EVs from ovarian cancer ascites and we also found that this protein was localized to AV-binding EVs (Figure 2(), lane 1). Furthermore, these AV-binding EVs carried more CD59 than AV-binding EVs from portal-hypertensive ascites (lane 5) after correcting for *β*-actin, making CD59 a potential biomarker for ovarian cancer.

Among the recently identified EV-based biomarkers for the diagnosis of ovarian cancer, EpCAM is one of the most intensively studied proteins. Several studies showed that the number of EpCAM-positive EVs in body fluids increases with disease progression [28–32]. However, in our EV isolations, EpCAM was not detectable (Figure 2). This could be due to the different isolation procedures used in the different studies, which result in different vesicle populations being analyzed, as well as highly variable contamination levels.

A well known protein which is overexpressed in many cancers is *β*-catenin. It acts via the Wnt/*β*-catenin signaling pathway promoting cell growth and proliferation [33]. A recent study showed that *β*-catenin can be transferred between cancer cells via EVs and promotes cell motility and proliferation in the recipient cells [34]. This protein was identified in all vesicle populations from ovarian cancer ascites (Figure 2(), lanes 1 to 3). Although the ratio of *β*-catenin to *β*-actin was higher in each of the three EVs in ovarian cancer ascites than those in the portal-hypertensive ascites, this ratio was similar to that in the no-ligand control suggesting that the signal is likely to be nonspecific.

### 3.4. MMP9 Cargo of AV-Binding EVs

The gelatinases MMP2 and MMP9 have crucial functions in cancer progression and have been identified in ovarian cancer tissues, ascites, and patient's circulation [35–37]. Using zymography in which the presence of both gelatinases in their active and inactive proform can be examined, we observed that relative to the portal-hypertensive ascites, the pooled ascitic fluid of ovarian cancer patients had a higher level of pro-MMP2 and pro-MMP9 with the molecular weights of 72 kDa and 92 kDa, respectively. Additionally, the pooled portal-hypertensive ascites had the 60 kDa active MMP2 which was not detected in ovarian cancer ascites (Figure 2(b), lane 1 versus 6). The higher MMP-levels in malignant ascites are generally consistent with the often reported higher expression and secretion of MMPs in the cancer microenvironment [38].

MMPs have already been reported to be present in EVs from ovarian cancer ascites and had been associated with a proinvasive effect on ovarian cancer cells [30, 39]. Here, we not only confirmed the presence of MMP9 in EVs but we also further demonstrated that the enzyme was present in its inactive proform and was restricted specifically to the AV-binding EVs (Figure 2(b)). This enzyme was not present in the CTB- or STB-EVs or in any of the three EV types isolated from the portal-hypertensive ascites. In addition to pro-MMP9 at 92 kDa, we observed two higher molecular weight MMPs at >200 kDa and 125 kDa in the AV-binding EVs from pooled ovarian cancer ascites. We hypothesized that the former could be multimers of MMP9 [40], while the latter could be a complex of MMP9 and neutrophil gelatinase B-associated lipocalin (NGAL) [40], which has been described in AV-binding EVs secreted by activated neutrophils [41].

**Figure 3:**
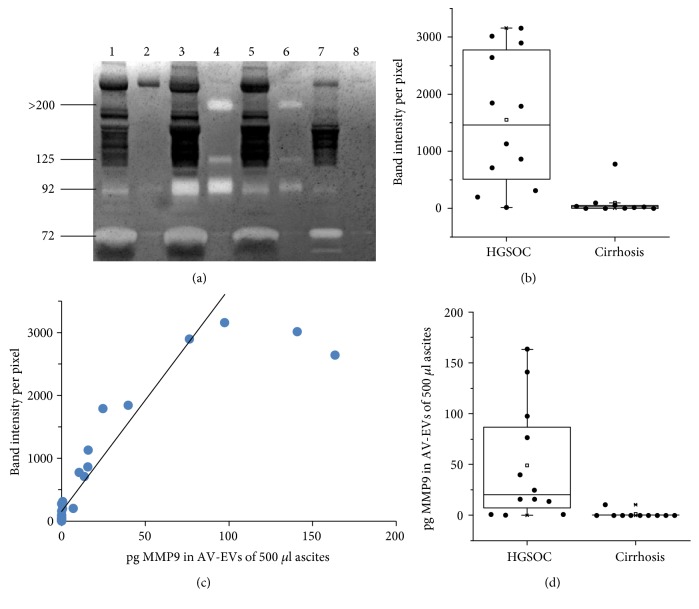
MMP9 presence and protein-level in AV-binding EVs from ascites. (a) Representative image of a zymogram to determine the presence of MMP9 in ascites samples from four individuals, where 1 *μ*l of ascites (lanes 1, 3, 5, and 7) and AV-binding EVs from 500 *μ*l ascites (lanes 2, 4, 6, and 8) were loaded. The sample in lanes 1 and 2 is from a patient with carcinosarcoma, the samples in lanes 3, 4, and 5, 6 are each from patients with HGSOC, and the sample in lanes 7 and 8 is from a patient with cirrhosis. (b) Box plot showing the difference in MMP9 band intensity analyzed by zymography in AV-EVs isolated from different ascites samples. (c) Correlation graph of MMP9-levels measured with zymography (*y*-axis) and ELISA (*x*-axis). (d) Box plot showing the difference in MMP9 amount analyzed by ELISA in AV-EVs isolated from different ascites samples (HGSOC = high-grade serous ovarian cancer).

To rule out any anomalies caused by the pooling of ascitic fluids, we analyzed ascites samples individually with zymography. Interestingly, we did not detect MMP9 in the AV-EVs from the ascites of patients with carcinosarcoma or clear cell histology. Instead, MMP9 was detected in AV-binding EVs of patients with high-grade serous ovarian cancer (HGSOC) (Figure 3(a)). The analysis of the gelatinolytic band intensities revealed that MMP9-levels were significantly higher in AV-EVs from HGSOC ascites compared to ascites AV-EVs from patients with cirrhosis (*p* = 6 × 10^−4^) (Figure 3(b)).

For further evaluation of this finding, the MMP9 concentration in AV-binding EVs was quantified by a sandwich ELISA. This was performed, because the detection range by zymography is very narrow. As shown in Figure 3(c), the band intensities in zymography only correlated with MMP9-levels up to 100 pg MMP9 per 500 *μ*l ascites at Spearman's rho of 0.885 (*p* = 1.85 × 10^−9^). Based on the ELISA analysis, MMP9-levels in AV-EVs were statistically significantly higher in patients with HGSOC, when compared to patients with cirrhosis (*p* = 3 × 10^−4^) (Figure 3(d)).

**Figure 4:**
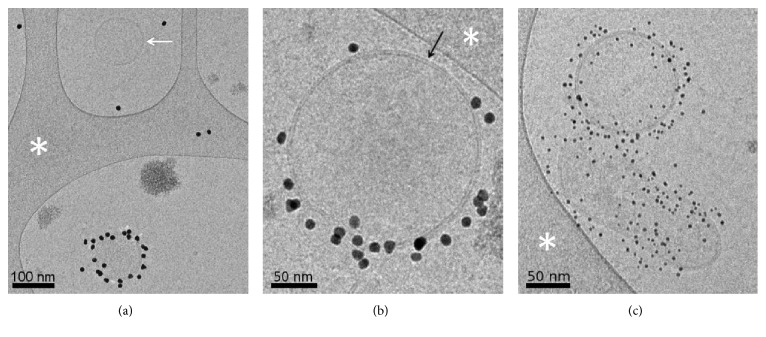
Cryo-EM images of individual EVs from the ascites of patients with HGSOC (a), carcinosarcoma (b), or liver cirrhosis (c). The samples were labeled with AV-conjugated gold nanoparticles (10 nm diameter in (a) and (b), 4 nm diameter in (c)). In (a), a non-AV-binding EV (white arrow) is seen, next to an AV-labeled EV. The lipid bilayer surrounding EVs is clearly resolved by cryo-EM (black arrow in (b)). The white asterisks point to areas of the carbon support film. Scale bars: 100 nm in (a) and 50 nm in (b) and (c).

To confirm that the lack of detectable MMP9 in AV-binding EVs from portal-hypertensive ascites was not due to the absence of AV-EVs, we examined ascites by the labeling of EVs with AV-conjugated gold nanoparticles and imaging with cryo-EM [15]. In all of the examined samples, including those that did not have detectable MMP9 in AV-EVs, both AV-binding EVs and non-AV-binding EVs were observed (Figure 4). In addition, both EV populations exhibited the characteristic lipid bilayer with a size range characteristic of EVs, that is, 50 to 500 nm in diameter [3].

As mentioned in the introduction, one of the greatest obstacles for the usage of EVs as biomarker in clinical applications is the lack of isolation and analysis methods that reliably and reproducibly purify and identify distinct populations of EVs, because contaminants like protein aggregates or lipoproteins that are abundantly present in body fluids are often copurified and cannot be discriminated from EVs. Consequently, the identification of distinct and clearly defined vesicle populations that harbor potential biomarkers is required for a successful clinical application. The AV-binding and MMP9-carrying EVs that were detected in HGSOC patients are candidates for utilization as ovarian cancer marker, because this EV population is defined by a combination of lipid and protein markers, which enhances the ability of specific purification and detection from complex body fluids. Even though these EVs were identified in ascites, they have potential as circulating marker, because of the constant exchange of fluids and molecules between the peritoneal cavity, the lymphatic system, and the blood stream [42, 43].

## 4. Conclusion

In summary, we demonstrated here a proof of concept that using membrane lipid-binding ligands to isolate different EV subpopulations from ascites fluid is an efficient method for biomarker discovery as it will enhance the signal-to-noise ratio. Specifically, by using ascites from small patient groups with HGSOC or cirrhosis and a small set of cancer-associated proteins, we observed that EV-associated MMP9 in HGSOC is predominantly localized in AV-binding EVs and that both CD59 and MMP9 in AV-binding EVs are candidate biomarkers for HGSOC. Without fractionating the EVs into subpopulations, MMP9 was unlikely to be identified as a candidate HGSOC biomarker as the high level of MMP9 in AV-EVs would have been obscured by the lack of MMP9 in other EVs. The utility of CD59 and MM9 in AV-binding EVs as ovarian cancer biomarker will have to be further evaluated in large patient cohort studies for their specificity and sensitivity.

## Figures and Tables

**Table 1:** Clinical characteristics of ovarian cancer patients; na = not available; ∗ indicates that these samples were only used in the pool, because the detailed clinical characteristics were not available.

Patient number	Histological type	Age at diagnosis	FIGO stage at diagnosis	Grade at diagnosis
1	Carcinosarcoma	na	IIIC	3
2	Serous papillary	60	IV	3
3	Serous papillary	52	IV	3
4	Serous papillary	46	IIIC	3
5	Carcinosarcoma	na	IIIC	3
6	Serous papillary	67	IV	2
7	Serous papillary	83	na	2
8	Serous papillary	60	IV	2
9^∗^	na	na	na	na
10	Serous papillary	39	IV	3
11	Serous papillary	70	na	3
12^∗^	na	na	na	na
13	Serous papillary	73	IIIC	3
14	Serous papillary	63	IIIC	2
15	Serous papillary	60	IIIC	3
16	Serous papillary	62	IIIC	3
17	Clear cell	61	IIIC	3
